# Supplementary material for: Hallucination of closed repeat proteins containing central pockets
Source: Nat Struct Mol Biol. 2023 Sep 28;30(11):1755–60. doi: 10.1038/s41594-023-01112-6 (PMC10643118; doi:10.1038/s41594-023-01112-6)
Supplement: Supplementary file 1 — Supplementary Figures 1 and 2 and Supplementary Table 1. [file 41594_2023_1112_MOESM1_ESM.pdf]

nature structural & molecular biology

Article

https://doi.org/10.1038/s41594-023-01112-6

Hallucination of closed repeat proteins containing central pockets

In the format provided by the authors and unedited

Supplementary Information
Hallucination of closed repeat proteins containing central pockets

Linna An[1,2]†*, Derrick R. Hicks[1,2]†, Dmitri Zorine[1,2]†, Justas Dauparas[1,2], Basile I. M. Wicky[1,2], Lukas F. Milles[1,2], Alexis Courbet[1,2,3], Asim K. Bera[1,2], Hannah Nguyen[1,2], Alex Kang[1,2], Lauren Carter[1,2], and David Baker[1,2,3]*

[1] Department of Biochemistry, University of Washington, Seattle, WA 98195, USA
[2] Institute for Protein Design, University of Washington, Seattle, WA 98195, USA.
[3] Howard Hughes Medical Institute, University of Washington, Seattle, WA 98195, USA.
* Corresponding author. Email: dabaker@uw.edu, linnaan@uw.edu
† These authors contributed equally

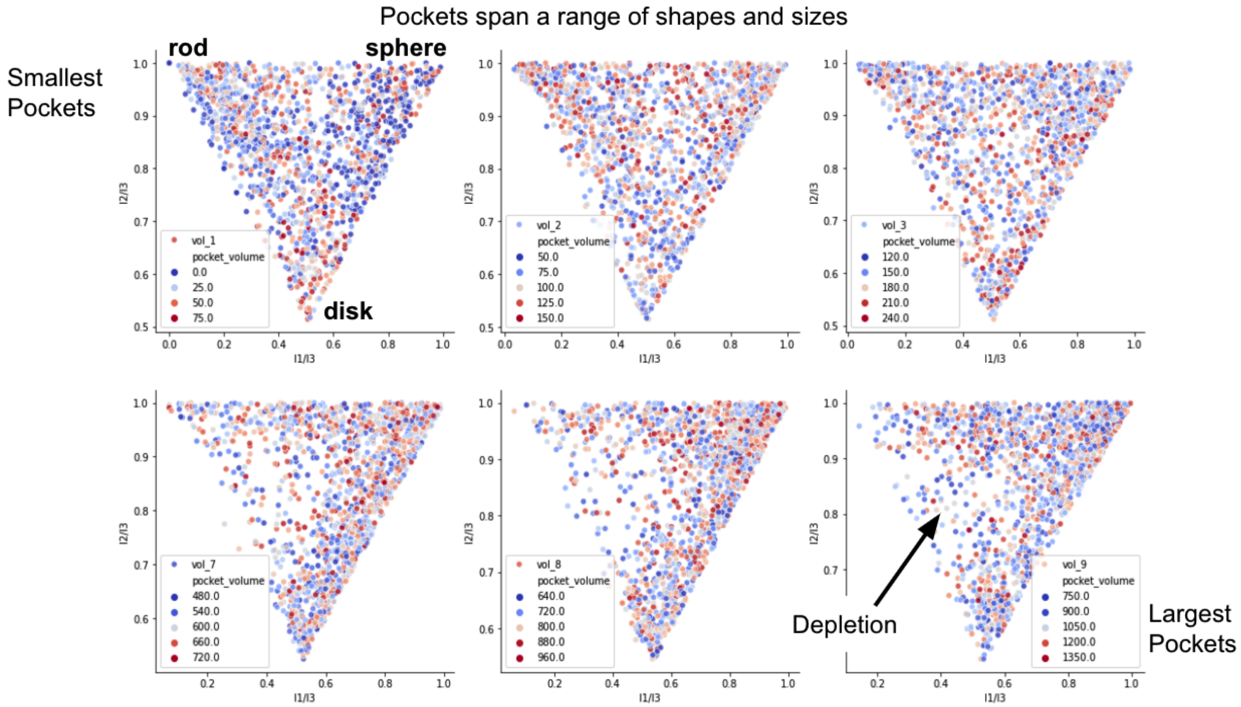

Figure S1. pocket shapes. Pockets were detected for 21,021 designs with sequences converted to poly-alanine to show max possible pocket size. Plots show the I2/I3 vs I1/I3 ratio which dictate the pocket shape. We show plots for pockets binned from small (75 cubic Å) to large (1400 cubic Å).

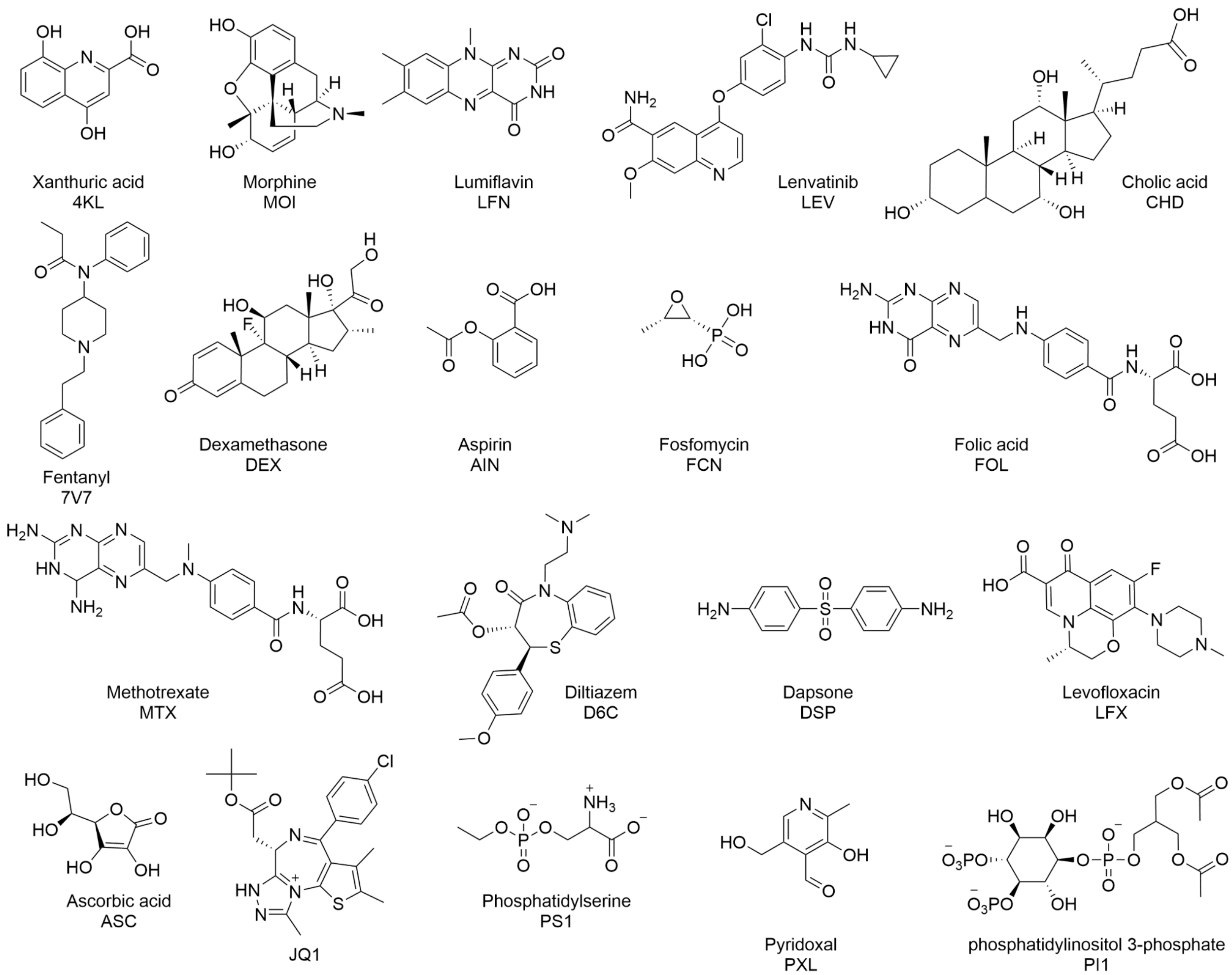

Figure S2. Ligand structures used for docking

The structure of ligands used for docking and design in this study. The three-letter names are marked out with the chemical name and structures.

Table S1. The sequence similarity of 96 experimentally-characterized pseudocycles to native proteins (UniRef50), and the structural similarity of 96 experimentally-characterized pseudocycles to native proteins in PDB by mTMalign.

	sequence similarity analysis							Structural similarity analysis			
Query	subject	%Identical match	alignment length	number of mismatch	number of gap openings	e_value	bit_score	Subject	Match Length	RMSD	TMscore
A1	UniRef50_A0A3S1AQK8	24.551	167	102	6	1.30E-05	51.6	2y2wF	108	2.43	0.555
A2	UniRef50_UPI001C447687	35.052	97	45	4	1.12E-04	48.5	3zbin	66	2.17	0.471
A3	UniRef50_UPI0012DD4EA6	37.736	106	52	6	1.54E-05	51.2	2m06A	104	1.44	0.805
A4	UniRef50_A0A419IXJ4	38.75	80	35	3	0.31	38.5	5upbC	72	2.19	0.535
A5	UniRef50_A0A6G6ZRG0	29.252	147	97	3	2.36E-05	51.6	5gpjB	133	2.11	0.771
A6	UniRef50_A0A7V7BPY5	28.205	156	96	6	0.052	42.4	4getC	99	2.43	0.511
A7	UniRef50_A0A175VRR7	26	150	93	3	5.52E-07	56.2	3rkoC	84	2.73	0.437
A8	UniRef50_A0A124FII4	28.834	163	65	6	9.56E-06	52	4j7cK	89	2.56	0.507
A9	UniRef50_A0A6A4TDX3	17.318	179	122	4	1.14E-04	50.1	5g04O	95	2.47	0.489
A10	UniRef50_UPI00067259DD	34.444	90	42	3	0.04	40.4	4pxdA	79	1.91	0.697
A11	UniRef50_A0A4C1WNI5	28.571	77	54	1	0.12	40.4	5yijA	79	2.49	0.406

A12	UniRef50_A0A227JIB8	28.333	120	74	4	0.59	38.5	2r4lA	84	2.65	0.486
B1	UniRef50_A0A6P7G917	42.5	80	41	4	0.16	38.9	4wmqA	58	2.73	0.393
B2	UniRef50_A0A3D5RHP1	26.119	134	90	3	1.00E-04	49.7	3dwoX	77	2.47	0.442
B3	UniRef50_A0A364NNE7	32.353	136	72	8	0.12	40.8	5kouB	64	2.38	0.349
B4	UniRef50_A0A850P5A7	32.353	34	23	0	0.14	37.7	1914A	78	2.49	0.454
B5	UniRef50_A0A127R2L3	24.545	110	76	3	0.12	40	6enyC	82	1.83	0.565
B6	UniRef50_UPI001BEAE40B	41.538	65	33	2	1	37.4	3jqoh	67	2.28	0.441
B7	UniRef50_A0A812C1J0	25.234	107	80	0	2.10E-04	48.5	2m06A	104	1.91	0.687
B8	UniRef50_A0A519V3C4	29.31	116	78	1	1.07E-05	52.4	2vhiC	83	2.57	0.444
B9	UniRef50_C5A613	26.923	182	80	4	1.53E-07	57.4	4getA	82	2.39	0.482
B10	UniRef50_A0A1S2LE79	30.657	137	82	4	0.004	44.7	5lg4C	71	2.04	0.425
B11	UniRef50_UPI0018DDE843	23.301	103	73	2	0.01	42.7	3g7gC	79	2.21	0.488
B12	UniRef50_UPI001901E18D	24.516	155	95	3	0.072	42	5injA	84	2.34	0.451
C1	UniRef50_A7S3P0	35.065	77	44	2	0.001	43.1	5m1bC	48	2.17	0.411

C2	UniRef50_A0A897ECV8	42.857	35	20	0	0.32	37.4	2f1tC	80	2.33	0.601
C3	UniRef50_V4B1W7	19.149	141	114	0	0.13	40.4	5lhpA	74	2.31	0.393
C4	UniRef50_A0A2V9J8M7	35.211	71	39	2	0.067	40	5halA	74	1.57	0.622
C5	UniRef50_A0A7L2CMC6	25.893	112	81	1	6.53E-05	48.5	1s7hD	74	1.86	0.539
C6	UniRef50_A0A8A7WM05	25.926	135	86	4	0.016	43.1	3pgsA	84	2.44	0.441
C7	UniRef50_A0A5C7GHG9	28.723	94	58	3	0.08	41.2	2h6oA	78	2.02	0.469
C8	UniRef50_A0A1X0ZNB1	34.783	69	41	3	0.012	43.9	4aklB	83	2.45	0.426
C9	UniRef50_A0A8F2N5L5	32.456	114	72	4	0.004	44.7	4d6jA	85	2.54	0.502
C10	UniRef50_UPI001AAC8935	41.667	108	47	4	8.74E-08	57.8	5kcgA	94	1.78	0.673
C11	UniRef50_A0A433UB03	35.955	89	57	0	9.37E-05	48.1	6os5C	88	1.57	0.768
C12	UniRef50_A0A8G1DBU1	37.5	64	40	0	0.096	38.1	3f1rA	54	1.67	0.619
D1	UniRef50_E9HZ43	32.039	103	49	6	1.42E-06	51.6	3gaqA	74	2.16	0.557
D2	UniRef50_A0A6J8BUN4	30.108	93	57	2	0.07	40.4	5upbC	68	2.43	0.464
D3	UniRef50_A0A2	31.765	85	49	3	0.011	41.6	4n7cA	70	1.91	0.617

	G8KM49										
D4	UniRef50_S4RYI5	38.462	117	55	5	0.006	42.7	2a8iA	79	2.23	0.51
D5	UniRef50_UPI0003F09BD3	36	75	45	1	0.028	41.2	5airB	67	1.94	0.522
D6	UniRef50_A0A5E8V3U9	35.338	133	86	0	4.54E-04	46.6	3eh3A	85	2.62	0.43
D7	UniRef50_A0A2J8B1B7	29.268	82	58	0	0.002	44.3	4getA	78	2.17	0.588
D8	UniRef50_A0A1L8GE06	21.429	112	83	1	1.67E-05	51.2	4maeB	79	2.48	0.468
D9	UniRef50_UPI001C447E7D	32.967	91	43	3	2.75E-05	49.7	2m06A	89	1.7	0.784
D10	No hits up to e_value <= 10	\	\	\	\	\	\	2lfuA	84	1.98	0.75
D11	UniRef50_A0A210Q9Q4	25.974	77	57	0	1.07E-05	49.7	4getA	74	2.1	0.565
D12	UniRef50_A0A2J8B1B7	34.375	96	62	1	3.27E-07	55.5	4getA	74	2.1	0.565
E1	UniRef50_A0A8C2PS63	37.037	162	85	10	2.15E-05	52	4tqoD	66	2.83	0.326
E2	UniRef50_A0A1G0X149	38.211	123	57	8	5.57E-05	49.7	1me5B	86	2.57	0.539
E3	UniRef50_A0A6A6SRW4	35.833	120	64	5	1.89E-05	50.8	5n6lB	71	2.35	0.441
E4	UniRef50_A0A410G1X1	36.792	106	50	4	0.011	42.7	4jm3A	72	1.92	0.567
E5	UniRef50_UPI0	21.6	125	76	2	0.12	40.8	2z2cA	81	2.41	0.463

	00A1CBA5D										
E6	UniRef50_A0A1I8IGK9	37.313	67	32	2	0.26	37.4	6i15A	65	20.9	0.634
E7	UniRef50_A0A0S4M5T5	37	100	49	4	0.002	45.8	1tmhC	86	2.48	0.515
E8	UniRef50_A0A6J8A802	29.268	123	75	3	4.99E-06	53.5	3wfbB	69	2.38	0.392
E9	UniRef50_A0A2G9R3R0	21.233	146	97	5	0.008	44.3	1n1zA	72	2.66	0.37
E10	UniRef50_A0A210Q9Q4	22.785	79	61	0	5.43E-07	53.1	2lfuA	83	1.94	0.747
E11	UniRef50_A0A1J4KZB5	36.735	49	24	2	0.26	37	5j9hA	58	1.71	0.706
E12	UniRef50_UPI0008736896	35.714	126	63	4	0.015	43.5	6qxaB	132	2.12	0.747
F1	UniRef50_A0A2C9JKN9	34.884	86	56	0	0.011	43.1	5m1bB	63	2.18	0.427
F2	UniRef50_A0A553I2W1	38.462	65	32	2	0.01	40.4	3snvA	62	2.33	0.568
F3	UniRef50_A0A2U3CKW1	28.431	102	66	2	0.05	42.4	6g1pB	81	2.3	0.426
F4	UniRef50_A0A0G4HSA4	31.481	162	82	7	8.1	35.4	5xdxA	82	2.49	0.428
F5	UniRef50_A0A3N0F6N4	29.6	125	75	4	0.001	46.2	1qftA	89	2.15	0.542
F6	UniRef50_A0A0C4WRM9	38.71	62	36	1	0.067	39.7	3l1sA	61	1.86	0.574

F7	UniRef50_A0A838EKD4	29.703	101	69	2	0.004	44.7	6nsjF	113	2.38	0.71
F8	UniRef50_A0A812SSI1	26.19	126	92	1	6.88E-06	52.4	1fftF	65	2.51	0.382
F9	UniRef50_A0A161TEC3	29.688	128	75	5	0.023	43.1	6os6A	69	2.58	0.35
F10	UniRef50_UPI0002F1B346	40	90	36	5	0.58	37	5od1A	83	1.32	0.802
F11	UniRef50_A0A101H2S0	37.097	62	37	2	0.18	39.7	1lf9A	77	2.08	0.527
F12	UniRef50_A0A842XWS7	32.143	84	50	3	0.11	40	2qqpG	76	2.2	0.513
G1	UniRef50_A0A1F7PMS7	34.426	61	35	2	0.091	38.9	5aj3J	69	1.59	0.718
G2	UniRef50_A0A6C0JML0	46.809	47	25	0	0.036	39.3	6p25A	67	1.89	0.437
G3	UniRef50_X1FW44	32.54	126	71	5	6.12E-04	47.4	2prrK	74	2.07	0.432
G3	UniRef50_X1FW44	32.432	74	47	1	4.4	36.2	3ag2A	66	2.51	0.409
G4	UniRef50_A0A6C2U5M5	30.612	98	56	2	8.43E-05	48.9	5lq4A	81	2.07	0.589
G5	UniRef50_A0A385PM13	20	100	64	2	0.22	38.9	6nsjE	107	2.01	0.716
G6	UniRef50_A0A661CIC3	28.44	109	76	2	2.11E-05	50.8	5ol6A	53	1.66	0.576
G7	UniRef50_A0A1F3JJD0	40.426	47	25	1	0.095	38.5	1ad1B	75	2.37	0.533

G8	UniRef50_A2EJ46	32.609	92	59	3	0.028	41.2	1ad1B	75	2.37	0.533
G9	UniRef50_UPI001AE9B6E4	31.461	89	60	1	2.73E-04	47.4	1fojB	63	1.9	0.468
G10	UniRef50_A0A2V5IZ99	38.028	71	41	2	0.15	38.1	2yfbB	84	1.36	0.868
G11	UniRef50_UPI001AE9B6E4	26.606	109	80	0	3.19E-05	50.1	1d0cB	63	1.93	0.465
G12	UniRef50_UPI00189C0A4D	40	40	24	0	1.4	36.2	2yjlC	72	2.08	0.613
H1	UniRef50_UPI0019D0A724	31.461	89	57	2	0.26	38.1	2g0jC	76	2.13	0.607
H2	UniRef50_A0A2D6KTY1	42	50	25	1	1.4	35.8	2ddzA	80	2	0.595
H3	UniRef50_A0A838N6Y5	32.955	88	53	2	0.018	40.8	6cwrA	72	1.83	0.615
H4	UniRef50_A0A543PA53	34.783	69	45	0	1.5	34.3	2yfbB	84	1.37	0.868
H5	UniRef50_A0A7S2VW98	39.623	53	31	1	5.3	34.7	5fq8G	75	2.28	0.573
H6	UniRef50_UPI0018FF684C	45.714	70	36	2	9.84E-06	48.9	3malB	64	2.04	0.632
H7	UniRef50_UPI00C290277	40.351	57	34	0	0.05	40.4	4fb2C	68	2.2	0.464
H8	UniRef50_A0A7J3MLV1	42	50	21	1	0.15	38.5	4p10A	63	1.94	0.522

H9	UniRef50_UPI001CD1A290	31.325	83	42	4	0.35	39.3	3v94G	81	2.3	0.469
H10	UniRef50_UPI001BB08C87	34.951	103	61	3	0.14	40	1u7zC	65	2.53	0.383
H11	UniRef50_E3MML6	27.737	137	78	4	0.057	42	1xw2A	73	2.62	0.368
H12	UniRef50_A0A0C9UQD2	31.481	108	65	3	0.009	42.7	5oxfC	87	2.21	0.649
