# Supplementary material for: Hallucination of closed repeat proteins containing central pockets
Source: Nat Struct Mol Biol. 2023 Sep 28;30(11):1755–60. doi: 10.1038/s41594-023-01112-6 (PMC10643118; doi:10.1038/s41594-023-01112-6)
Supplement: Supplementary file 2 — Reporting Summary [file 41594_2023_1112_MOESM2_ESM.pdf]

nature portfolio

Corresponding author(s): Linna An*, Derrick R. Hicks*, Dmitri Zorine*, Justas Dauparas, Basile I. M. Wicky, Lukas F. Milles, Alexis Courbet, Asim K. Bera, Hannah Nguyen, Alex Kang, Lauren Carter, and David Baker

Last updated by author(s): Jan 9, 2023

Reporting Summary

Nature Portfolio wishes to improve the reproducibility of the work that we publish. This form provides structure for consistency and transparency in reporting. For further information on Nature Portfolio policies, see our Editorial Policies and the Editorial Policy Checklist.

Statistics

For all statistical analyses, confirm that the following items are present in the figure legend, table legend, main text, or Methods section.

n/a	Confirmed	
☐	☒	The exact sample size (*n*) for each experimental group/condition, given as a discrete number and unit of measurement
☐	☒	A statement on whether measurements were taken from distinct samples or whether the same sample was measured repeatedly
☒	☐	The statistical test(s) used AND whether they are one- or two-sided<br>*Only common tests should be described solely by name; describe more complex techniques in the Methods section.*
☐	☒	A description of all covariates tested
☒	☐	A description of any assumptions or corrections, such as tests of normality and adjustment for multiple comparisons
☐	☒	A full description of the statistical parameters including central tendency (e.g. means) or other basic estimates (e.g. regression coefficient) AND variation (e.g. standard deviation) or associated estimates of uncertainty (e.g. confidence intervals)
☒	☐	For null hypothesis testing, the test statistic (e.g. *F*, *t*, *r*) with confidence intervals, effect sizes, degrees of freedom and *P* value noted<br>*Give P values as exact values whenever suitable.*
☒	☐	For Bayesian analysis, information on the choice of priors and Markov chain Monte Carlo settings
☒	☐	For hierarchical and complex designs, identification of the appropriate level for tests and full reporting of outcomes
☒	☐	Estimates of effect sizes (e.g. Cohen's *d*, Pearson's *r*), indicating how they were calculated

Our web collection on statistics for biologists contains articles on many of the points above.

Software and code

Policy information about availability of computer code

Data collection	The rosetta macromolecular modeling suite (www.roseuacornmons.org) is freely avaliable to non-commercial users. The model of all unique scaffolds (9838) and the sequences and the design models of the 96 characterized designs were uploaded to github (https://github.com/LAnAlchemist/Psedocycles_NSMB.git). The scaffold generation scripts were uploaded to github (https://github.com/dmitropher/af2_multistate_hallucination.git).
Data analysis	The crystallography data were analyzed using COOT. The circular dichroism and size exclusive chromatography data were analyzed using in-house python scripts.

For manuscripts utilizing custom algorithms or software that are central to the research but not yet described in published literature, software must be made available to editors and reviewers. We strongly encourage code deposition in a community repository (e.g. GitHub). See the Nature Portfolio guidelines for submitting code & software for further information.

Data

Policy information about availability of data

All manuscripts must include a data availability statement. This statement should provide the following information, where applicable:

- Accession codes, unique identifiers, or web links for publicly available datasets
- A description of any restrictions on data availability
- For clinical datasets or third party data, please ensure that the statement adheres to our policy

All the raw SEC, SAXS data can be provided upon request, all the other types of data have been made available to all. Data deposition, atomic coordinates, and structure factors reported in this paper were deposited in the PDB (PDB ID 8FJE for E8, 8FJF for H10 and 8FJG for H12). The Rosetta macromolecular modeling suite (http://www.rosettacommons.org) is freely available to academic and non-commercial users. The biochemical and biophysical characterization of the designs, structure prediction, sequence analysis, and X-ray crystallography statistics are provided as Supplementary Figures and Tables. The AlphaFold Protein Structure database used for structural analysis is freely available (https://alphafold.ebi.ac.uk).

Research involving human participants, their data, or biological material

Policy information about studies with human participants or human data. See also policy information about sex, gender (identity/presentation), and sexual orientation and race, ethnicity and racism.

Reporting on sex and gender	N/A
Reporting on race, ethnicity, or other socially relevant groupings	N/A
Population characteristics	N/A
Recruitment	N/A
Ethics oversight	N/A

Note that full information on the approval of the study protocol must also be provided in the manuscript.

Field-specific reporting

Please select the one below that is the best fit for your research. If you are not sure, read the appropriate sections before making your selection.

☒ Life sciences ☐ Behavioural & social sciences ☐ Ecological, evolutionary & environmental sciences

For a reference copy of the document with all sections, see nature.com/documents/nr-reporting-summary-flat.pdf

Life sciences study design

All studies must disclose on these points even when the disclosure is negative.

Sample size	96 designed proteins were selected for expression, the sample size was determined by the representation of the designed dataset and the availability of the resources.
Data exclusions	None of the molecular biology data from the 96 designed proteins were excluded.
Replication	The proteins were purified multiple times to get enough material for different experiments such as crystallography or Small-angle X-ray scattering.
Randomization	No randomization is needed in any of our molecular biology experiments.
Blinding	No blinding is needed in any of our molecular biology experiments.

Reporting for specific materials, systems and methods

We require information from authors about some types of materials, experimental systems and methods used in many studies. Here, indicate whether each material, system or method listed is relevant to your study. If you are not sure if a list item applies to your research, read the appropriate section before selecting a response.

Materials & experimental systems

n/a	Involved in the study
☒	☐ Antibodies
☒	☐ Eukaryotic cell lines
☒	☐ Palaeontology and archaeology
☒	☐ Animals and other organisms
☒	☐ Clinical data
☒	☐ Dual use research of concern
☒	☐ Plants

Methods

n/a	Involved in the study
☒	☐ ChIP-seq
☒	☐ Flow cytometry
☒	☐ MRI-based neuroimaging
